# Genome-Wide Association Study on Total Starch, Amylose and Amylopectin in Barley Grain Reveals Novel Putative Alleles

**DOI:** 10.3390/ijms22020553

**Published:** 2021-01-07

**Authors:** Mengdi Li, La Geng, Shanggeng Xie, Dezhi Wu, Lingzhen Ye, Guoping Zhang

**Affiliations:** 1 Institute of Crop Science, Zhejiang University, Hangzhou 310058, China; limengdi@zju.edu.cn (M.L.); 21816022@zju.edu.cn (L.G.); sgxie@zju.edu.cn (S.X.); wudezhi@zju.edu.cn (D.W.); zhanggp@zju.edu.cn (G.Z.); 2 Shandong (Linyi) Institute of Modern Agriculture, Zhejiang University, Linyi 276000, China

**Keywords:** *Hordeum vulgare* L., total starch (TS), amylose (AC), amylopectin (AP), core collection, genome-wide association study (GWAS), genotypic difference, quantitative trait loci (QTLs)

## Abstract

The content and composition of starch in cereal grains are closely related to yield. Few studies have been done on the identification of the genes or loci associated with these traits in barley. This study was conducted to identify the genes or loci controlling starch traits in barley grains, including total starch (TS), amylose (AC) and amylopectin (AP) contents. A large genotypic variation was found in all examined starch traits. GWAS analysis detected 13, 2, 10 QTLs for TS, AC and AP, respectively, and 5 of them were commonly shared by AP and TS content. qTS-3.1, qAC-6.2 and qAP-5.1 may explain the largest variation of TS, AC and AP, respectively. Four putative candidate genes, i.e., *HORVU6Hr1G087920*, *HORVU5Hr1G011230*, *HORVU5Hr1G011270* and *HORVU5Hr1G011280*, showed the high expression in the developing barley grains when starch accumulates rapidly. The examined 100 barley accessions could be divided into two groups based on the polymorphism of the marker S5H_29297679, with 93 accessions having allele GG and seven accessions having AA. Moreover, significantly positive correlation was found between the number of favorable alleles of the identified QTLs and TS, AC, AP content. In conclusion, the identified loci or genes in this study could be useful for genetic improvement of grains starch in barley.

## 1. Introduction

Barley (*Hordeum vulgare* L.) ranks the fourth largest cereal crop in term of planting area worldwide, only after maize, rice and wheat (FAOSTAT 2018). Like other cereals, the main composition in grains of barley is carbohydrate, mainly starch, which occupies approximately 62–77% of total dry weight [1,2]. Starch in barley grains consists of amylose and amylopectin, and their quantity and proportion have a great impact on nutrition and taste quality when barley is used as food, and on malt quality when used as raw material in brewing industry [3,4,5]. According to amylose content, barley can be classified into waxy type (below 5% amylose), high-amylose type (>35% amylose) and normal type (5–35% amylose) [6]. In general, most barley varieties belong to normal type. A study on 254 spring barley cultivars showed that amylose and amylopectin content varied from 16.74% to 30.92% and 26.47% to 42.97%, respectively [6]. While another study by using 39 barley cultivars collected from the diverse regions found four waxy cultivars with amylose content of 2.5–8.3% [7]. On the whole, the genetic variation of amylose and amylopectin content in barley grains has been less investigated.

The synthesis of amylose is controlled by *Waxy* gene encoding granule-bound starch synthase I (GBSSI), while synthesis of amylopectin is regulated by several relevant enzymes, including soluble starch synthase (SSS), starch branching enzyme (SBE), starch debranching enzyme (DBE), and glucan water dikinase (GWD) [8]. These three key enzymes play essential roles in amylopectin branching, debranching and phosphorylation and ensure the formation of proper molecular structures. It has been reported that precise editing of *Wx* gene resulted in the alteration of amylose content and kernel characteristics [9]. The *sex6* mutants lack starch synthase IIa (*ssIIa*) activity resulted a decreased amylopectin content, shortened chain length distribution and reduced gelatinization temperature [10]. The starch-branching enzyme I (*sbe1*) mutant exhibited apparently altered structure of amylopectin [11]. The *sugary* mutant is defective in starch debranching enzyme I (*dbe I*), therefore affecting amylopectin biosynthesis [12]. Overexpression potato GWD increased starch-bound phosphate content in barley caryopsis starch [13]. Although above-mentioned synthetic enzyme genes are related to starch properties, content and proportion of starch are also controlled by many regulators. For example, the rice starch regulator1 (RSR1), an APETALA2/ethylene-responsive element, regulate amylose content and amylopectin structure [14]. So far such regulators are rarely investigated in barley. In short, biosynthetic process of starch is quite complicated and tuned finely. Therefore, it is imperative to identify these regulators in order to elucidate starch biosynthesis process in barley. 

Genome-wide association study (GWAS) is an effective method for identifying the genes or loci controlling the sophisticated characters, based on phenotypes and genotypes association [15,16]. GWAS analysis has been performed on many complicated traits in barley, including salinity tolerance [17], drought tolerance [18], spot blotch (SB) resistance [19], malting quality [20] and agronomical traits [21]. In the present study, we take advantage of GWAS-based methodology for identifying starch-related genes or loci in barley, using 100 accessions from International Barley Core Selected Collection. As a result, several significant QTLs controlling total starch, amylose and amylopectin content of barley grains were identified.

## 2. Results and Analysis

### 2.1. Phenotypic Variation of Starch Traits

**Figure 1:**
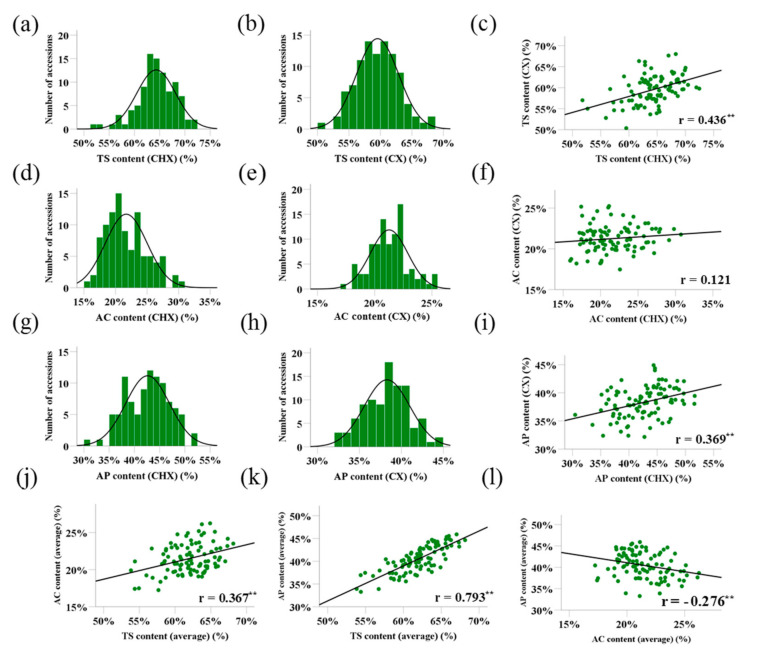
Frequency distribution and correlation analysis of TS, AC and AP content. (**a**,**b**) the frequency distribution of TS in Changxing and Cixi; (**c**) the correlation of TS between Changxing and Cixi; (**d**,**e**) the frequency distribution of AC in Changxing and Cixi; (**f**) the correlation of AC between Changxing and Cixi; (**g**,**h**) the frequency distribution of AP in Changxing and Cixi; (**i**) the correlation of AP between Changxing and Cixi; (**j**) the correlation of TS and AC; (**k**) the correlation of TS and AC; (**l**) the correlation of AC and AP. TS, Total starch content; AC, Amylose content; AP, Amylopectin content. ** representing significance at *p* < 0.01 level, respectively (two-tailed).

In order to clarify the genotypic variation of the starch traits in barley grains, we analyzed total starch content (TS), amylose (AC) and amylopectin content (AP) in 100 accessions from the International Barley Core Selected Collection (BCS) [22] under two different environments. The results showed that the contents of all three starch traits were normally distributed over the population of the 100 barley accessions (Figure 1), indicating these starch traits in barley grains are quantitative characters, controlled by multi-genes. The contents of TS, AC and AP ranged from 50.36% to 72.46%, 15.93% to 30.73% and 30.47% to 51.64% in the 100 barley accessions, respectively (Figure 1; Table 1). ANOVA analysis showed significant differences among genotypes for the three starch traits (*p* < 0.001), and the genotype could explain 43.50%, 48.06% and 43.59% of total variation for TS, AC and AP contents, respectively (Table S2). In addition, significantly positive correlations were found for TS and AP between the two environments (Figure 1c,i). The heritabilities (h^2^) of TS and AP were 67.44% and 52.79%, respectively. However, there is no significant correlation for AC between the two environments (Figure 1f), indicating that it is largely influenced by environment. Moreover, there were significantly positive correlation between TS and AC (Figure 1j), TS and AP (Figure 1k), and significantly negative correlation between AC and AP (Figure 1l). 

### 2.2. Genome-Wide Association Mapping

To explore the genetic factors associated with the starch content, a GWAS analysis was conducted by using starch content data and SNPs of 100 BCS accessions. As a result, 191,147 out of 1,084,274 SNP markers were obtained with minor allele frequency (MAF) >5% and missing rate <15% filter criteria for further GWAS analysis. The average marker density was 0.0396 SNP per kilobase (Table S3).

**Figure 2:**
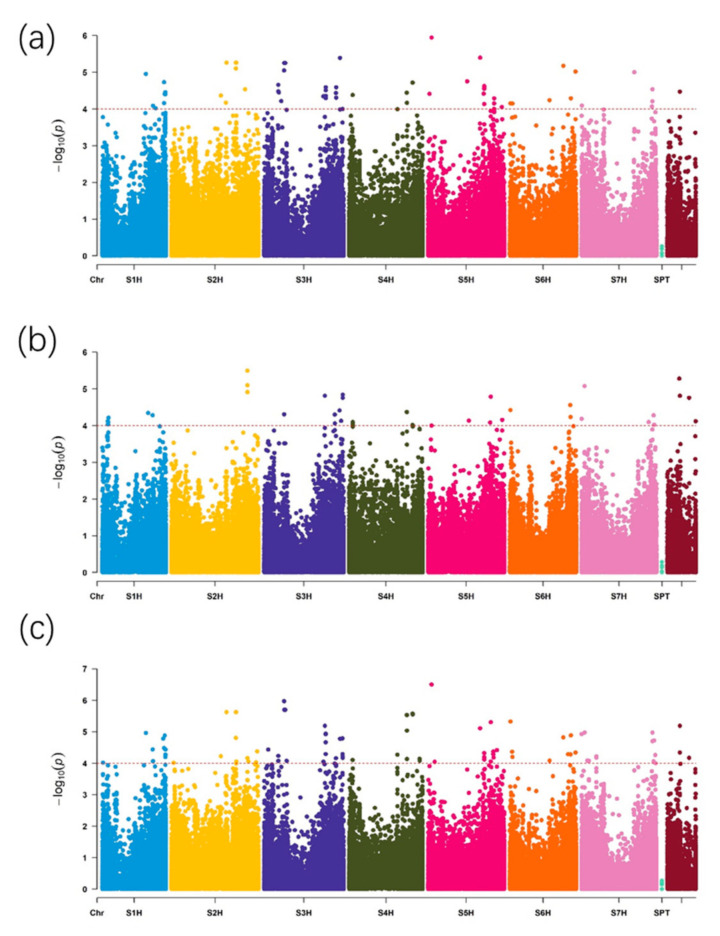
Manhattan plots for amylopectin content. (**a**) Manhattan plot of E1 (Changxing). (**b**) Manhattan plot of E2 (Cixi). (**c**) Manhattan plot of the average data. Dashed line represents the significance threshold (*p* < 10^−4^).

Based on the genome-wide association mapping, with a significant level *p* < 10^−4^, 19, 18, and 16 QTLs were detected for TS in E1 (Changxing), E2 (Cixi) and E3 (average of E1 and E2); 8, 14, and 6 QTLs for AC and 18, 15, and 19 QTLs for AP, respectively (Table S4). These QTLs were distributed over the all 7 chromosomes of barley genome (Figure 2, Figures S2 and S4) (Table S4). The relationship between the observed and expected *p*-value can be illustrated by using QQ plots, which indictes that both false-positives and false-negatives were well controlled when the straight line is closed to the diagonal line but with a sharp upward deviated tail [23]. Our results showed that the predicted *p* value was closed to the actual value, indicating that the results were reliable and false-positive and false-negatives rate of the associated SNPs was low (Figure S2). 

QTLs identified in more than two environments were considered to be stable. Among the identified QTLs, 13, 2, and 10 stable QTLs were detected for TS, AC and AP contents, respectively (Table S4). Among these stable QTLs, the loci explaining the largest phenotypic variation of TS was qTS-3.1, which is located on chromosome 3H, explaining 27.15% of total variation in E3, and it was also detected in E1 and E2. The major QTL of AC was qAC-6.2, contributing to about 20% of the phenotypic variation. The major QTL of AP was qAP-5.1, associated with the marker S5H_29297679, explaining 25.7% of total variation in E3, and it could be also detected in E1 and E2. In addition, this QTL was also detected for TS in E3 (Table S4).

Among the above-mentioned stable QTLs, 5 QTLs were detected for both TS and AP. For example, qTS-2.1, a same locus as qAP-2.1, associated with marker S2H_484808981, was identified for both TS and AP, explaining 21.46% to 26.01% of phenotypic variation. Similarly, qTS-3.1 (qAP-3.1, S3H_176458677), qTS-6.2 (qAP-6.2, S6H_349173230), qTS-6.3 (qAP-6.3, S6H_469691793) and qTS-7.1 (qAP-7.1, S7H_26188860), were all significantly associated with both TS and/or AP, accounting for 20.92% to 27.15%, 15.57% to 21.18%, 17.52% to 25.98% and 16.68% to 21.25% of the phenotypic variation, respectively (Figure S5; Table 2).

### 2.3. Identification of the Candidate Genes Associated with Significant QTLs

**Figure 3:**
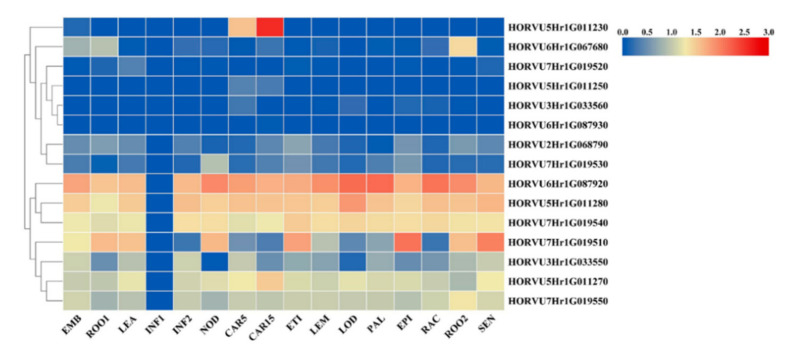
Heatmap of 15 candidate genes expression level in different tissues and developing stages. EMB: 4-day embryo, ROO1: Roots from seedlings (10 cm shoot stage), LEA: Shoots from seedlings (10 cm shoot stage); INF1: Young developing inflorescences (5 mm); INF2: Developing inflorescences (1–1.5 cm); NOD: Developing tillers, 3rd internode (42 DAP); CAR5: Developing grain (5 DAP); CAR15: Developing grain (15 DAP); ETI: Etiolated seedling, dark cond. (10 DAP); LEM: Inflorescences, lemma (42 DAP); LOD: Inflorescences, lodicule (42 DAP); PAL: Dissected inflorescences, palea (42 DAP); EPI: Epidermal strips (28 DAP); RAC: Inflorescences, rachis (35 DAP);ROO2: Roots (28 DAP). SEN: Senescing leaves (56 DAP).

Among three major QTLs and five common QTLs, a major QTL for TS was identical with a common QTL named qTS-3.1 (qAP-3.1). Based on the seven highly significant QTLs for TS, AC and/or AP, the putative candidate genes were searched within 100 kb upstream and downstream of the significant association markers. As a result, a total of 25 genes were detected within ±100 kb of these 7 QTLs. The transcript expression information of 25 candidate genes were obtained from barley genome database. After deleting the candidate genes with no expression during the whole growth stage, 15 candidate genes were used to draw the expression heatmap, indicating that the transcript level of these candidate genes varied greatly with the different tissues and development stages (Figure 3; Table S5). It’s worth noting that the gene *HORVU6Hr1G087920*, *HORVU5Hr1G011230*, *HORVU5Hr1G011270* and *HORVU5Hr1G011280*, associated with qAC-6.2 and qAP-5.1, showed the high expression in the developing grains during 5th to 15th days after pollination (DAP) (Figure 3), when starch accumulates rapidly in grains. Thus, it may be suggested that these four putative candidate genes are highly associated with starch accumulation in grains. 

**Figure 4:**
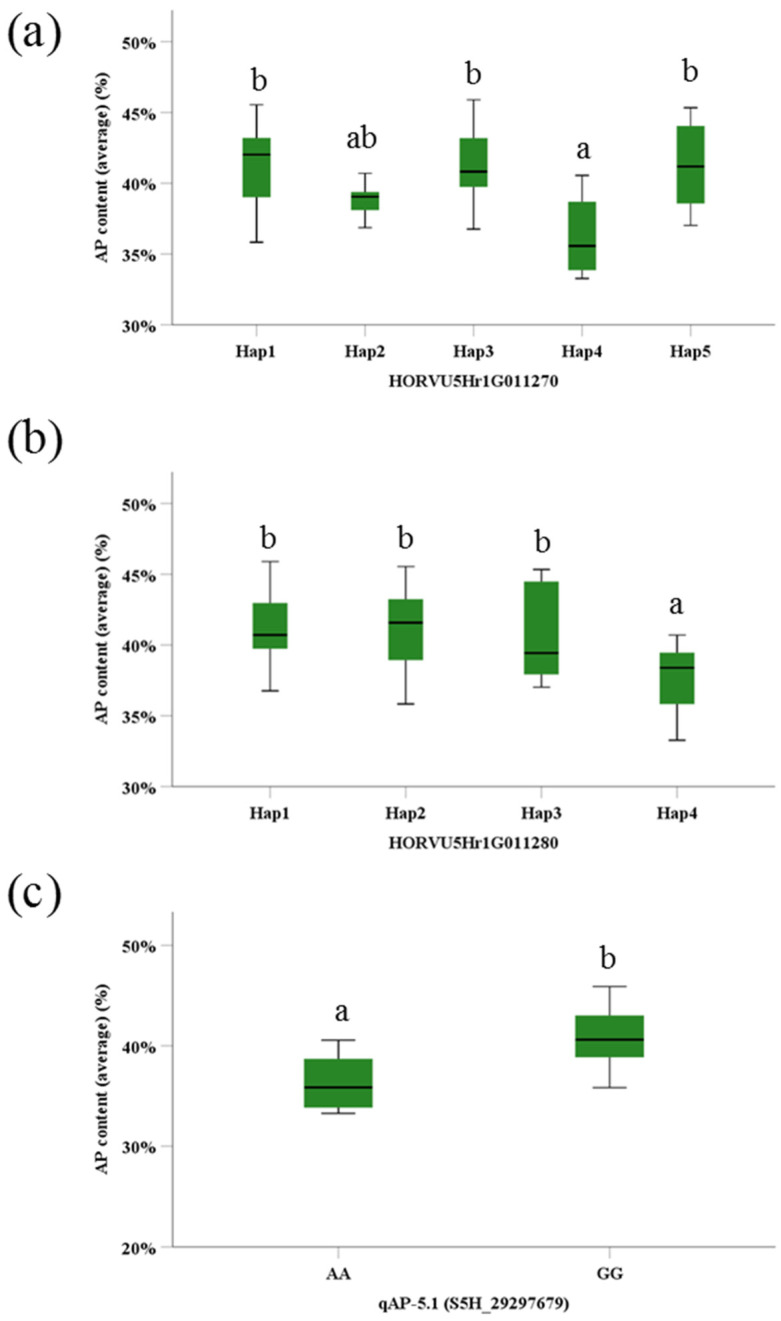
Haplotypes and Phenotypic differences of AP accumulation based on the largest explanation of phenotypic variation. (**a**) HORVU5Hr1G011270 resulted in five haplotypes depending on the polymorphism of 11 SNPs. (**b**) HORVU5Hr1G011280 resulted in four haplotypes depending on the polymorphism of 5 SNPs. (**c**) The average data of 100 barley accessions which was divided into two groups of AA and GG genotypes according to QTL qAP-5.1 (S5H_29297679). Different letters represent the significant differences (*p* < 0.05).

Haplotype analysis showed that *HORVU5Hr1G011270* and *HORVU5Hr1G011280*, two of the four candidate genes, contributed to highly significant difference in AP content (*p* < 0.01) (Figure 4a,b; Tables S6 and S7). The gene *HORVU5Hr1G011270* contains 11 SNPs polymorphisms, involving five haplotypes in 100 BSC collection (Table S6). The mean AP content of Hap4 was 36%, being significantly lower than that of other four hyplotypes. On the other hand, there were five SNPs polymorphisms for the gene *HORVU5Hr1G011280*, resulting in four hyplotypes (Table S7). The mean AP content of Hap4 was 38%, being significantly lower than that of other three hyplotypes. Moreover, these two genes were also the candidate genes of the same QTL (qAP-5.1, marker S5H_29297679), which is associated with AP content in barley grains. Among 100 barley accessions, two groups could be divided based on the polymorphism of the marker S5H_29297679; 93 accessions had allele GG and 7 accessions had AA for the locus. The polymorphism of this marker fits well with the phenotypic data (Figure 4c). The mean AP content of AA type was 32.95%, being significantly (*p* < 0.001) lower than that of GG type (40.79%). Moreover, this QTL was also associated with TS. Therefore, it may be suggested that this QTL and the associated candidate genes are important in determining AP content in barley grains. 

Moreover, we made a homology search of the genes related with starch traits, based on barley genome database. As a result, we mapped all the associated genes as well as the stable QTLs identified in this study in barley chromosomes (Figure S6). It could be seen that some genes are located at the similar positions as the identified QTLs on chromosomes, such as *AGPL1* vs. qTS-1.2 on 1H, while most of the identified QTLs are novel.

### 2.4. Effect of Favorable Alleles on Total Starch, Amylose and Amylopectin Content in Barley Grains

**Figure 5:**
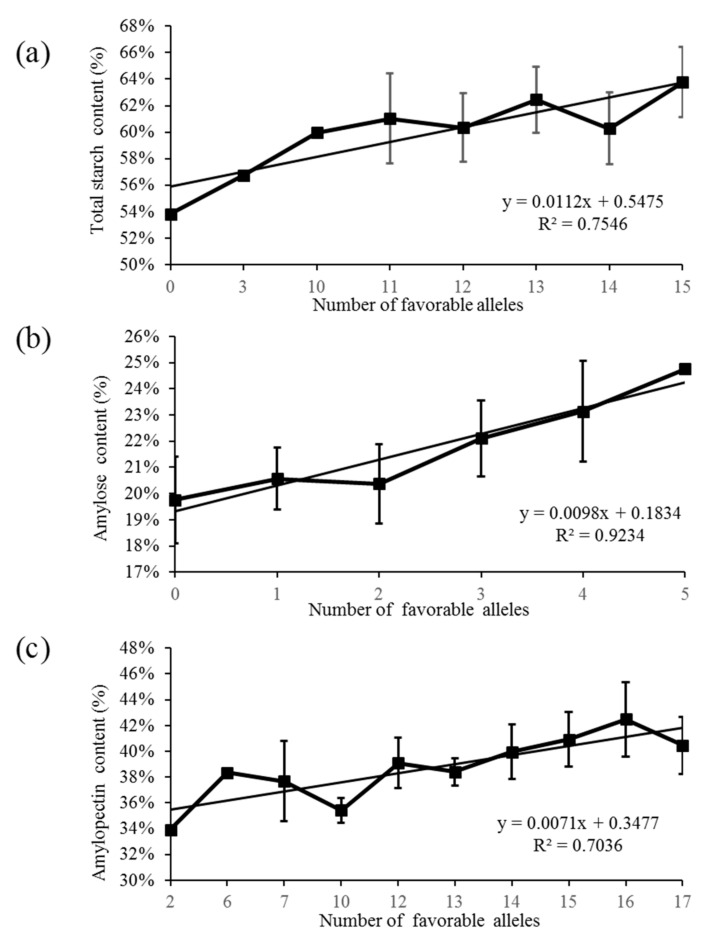
Linear regression analysis for the number of favorable alleles and starch content. (**a**), total starch content; (**b**), amylose content; (**c**), amylopectin content.

In order to clarify the breeding effect of the detected stable QTLs, we introduced a term so-called favorable allele. The allele, which could increase the contents of TS, AC or AP is defined as favorable allele. The number of favorable alleles ranged from 0 to 15 for TS, 0 to 5 for AC and 2 to 17 for AP (Table S8). Linear regression analysis was performed on the number of favorable alleles and three starch traits for the significant QTLs detected in E3. The results showed the significantly positive correlation between TS (r = 0.87, *p* < 0.001), AC (r = 0.96, *p* < 0.001) or AP (r = 0.84, *p* < 0.001) and the number of favorable alleles (Figure 5).

## 3. Discussion

### 3.1. Genetic Variation of Three Starch Traits among 100 BCS

The complex and sophisticated process of starch synthesis limited the selection of favorable accessions with desired amylose and/or amylopectin content. Moreover, it is still far from clear what function the amylose and amylopectin play in the determination of barley grain property and application. In this study, we performed a genome-wide association analysis of starch-related traits in barley grains by using 100 accessions collected from the International Barley Core Selected Collection (BCS) originated from 41 countries or regions. In contrast to single or few source of accessions [6,24], extensive sources ensure larger genotypic difference. This study revealed numerous novel QTLs associated with starch-related traits and identified several relevant genes and alleles, which could be useful in barley quality improvement. 

There is a large phenotypic variation of starch, amylose and amylopectin in the population of the 100 accessions, ranging from 50.36% to 72.46%, 15.93% to 30.73% and 30.47% to 51.64%, respectively (Figure 1; Table 1). However, no any waxy accession was found, as amylose content of all tested genotypes was higher than 5%. The examined 100 accessions in this study were collected from different areas in the world, representing a wide genetic diversity, so it may be suggested that waxy trait is very rare in barley. Shu & Rasmussen [6] did not find waxy barley genotype (amylose content <5%) in a collection of 254 European spring barley varieties. However, a higher proportion of waxy genotypes could be found in other cereal crops, such as rice and maize [25,26]. The rare waxy genotypes in barley might be attributed to its end-use, as non-waxy barley is preferred for use in beer and feed production. Actually in China many landrace and naked barley accessions planted in Tibet, which are used as food, belong to the waxy type. 

### 3.2. Functionally Dissection for Barley Grain AP Property Manipulation

Several attempts have been made to identify the loci controlling starch traits in barley [6,24]. Mohammadi et al. [24] reported that a SNP marker highly related with amylose content was obtained from *Wx* gene promoter. Shu & Rasmussen [6] claimed that in addition to the *Wx* loci, many other sites were also important for amylose content. In our study, being consistent with no any waxy genotype (AP < 5%) was detected in the 100 BCS, *Wx* gene located on chromosome 7H was not detected in the GWAS analysis. On the other hand, many novel minor genes and QTLs controlling starch traits were detected in this population with a large genetic diversity. AGPL1, one of the members of ADP-glucose pyrophosphorylase (AGPase), is close to qTS-1.2, which is responsible to starch accumulation (Figure S6). After converting glucose-1-phosphate to activated glucosyl donor ADP-glucose by AGPase, GBSS and SSS are involved in the synthesis of amylose and amylopectin, respectively [8]. In cereal crops, AC has been intensively concerned and investigated. It was reported that expression of waxy gene has significant influence on eating and cooking quality (ECQ) in rice [27], and malting quality and starch paste viscosity in barley [28,29]. In addition, regulation of *amo1* gene was also widely applied in AC manipulation in cereals [30,31]. However, little research has been done on AP associated genes. Amylose/amylopectin ratio has a great impact on the properties of cereal starches, including gelatinization, solubility and the formation of resistant starch (RS) [32,33]. Therefore, it is a great potential to modify starch composition by manipulating AP-associated genes. In this study, we identified 5 QTLs controlling both TS and AP, indicating that these loci or putative genes have polygenic effect, and should be greatly concerned in further study. 

In addition, we identified a major QTL (qAP-5.1, marker S5H_29297679) controlling AP accumulation. This QTL was also detected for TS in E3 (Table S3), suggesting it regulate AP and TS simultaneously. Moreover, it was found that two candidate genes (*HORVU5Hr1G011270* and *HORVU5Hr1G011280*) of qAP-5.1, which controls AP accumulation in grains, showed high expression in the developing grains at 5 and /15 DAP). *HORVU5Hr1G011270* is known as the gene encoding β-1,4-*N*-acetylglucosaminyltransferase family protein. Its homologous gene in *Arabidopsis thaliana* named *AT2G13290*, has the ability of transferring glycosyl groups according to the reference of GO biological process and molecular function. The biological process of glycosyl groups transfer has a great effect on the degradation and transformation of starch [34]. Thus *HORVU5Hr1G011270* might have a marked effect on starch synthesis via glycosyl groups transfer. On the other hand, *HORVU5Hr1G011280* is also associated with qAP-5.1 and annotated as Cullin-associated NEDD8-dissociated protein 1 (CAND1). Its homologous genes in *Arabidopsis* and rice have been studied in the functions. *AtCAND1* and *OsCAND1* play the key roles in auxin signaling and plant development [35,36]. while auxin signaling has great impact on endosperm development and starch synthesis [37,38]. In short, it may be suggested that *HORVU5Hr1G011270* and *HORVU5Hr1G011280* might be the key genes regulating starch construction and accumulation via glycosyl groups transfer and auxin signaling.

### 3.3. Potential Implications for Barley Breeding with Favorable Starch Content

The content and composition of starch in barley grains determine the yield, quality and end-use of barley, which is an important research and breeding object. In the cultivation and utilization of barley, the genotype selection of suitable starch content and composition is very important. In this study, the linear regression relationship were established between three starch traits content and the number of favorable alleles. Therefore, barley genotype with different starch content and composition can be directly selected according to the number of favorable alleles. 

Barley is the favorable material for malting and brewing industry. During the process of malting and mashing, the starch in barley grain are digested by hydrolytic enzymes intensively. Then in the brewing and fermentation stage, the digested starch were converted to alcohol by yeast. Therefore, the starch is the important trait in malting barley. However, few study was done to determine the relation between starch composition and malting quality. So, in the further work, we can use the specific materials and molecular markers identified in this study to screen and create new barley genotypes with specific starch content and composition, so as to study the relationship between starch traits and malting quality.

## 4. Materials and Methods

### 4.1. Plant Materials and Field Experiments

A collection of 100 barley (*Hordeum vulgare* L.) accessions (Table S1) selected from the BCS, including 34 two-rowed and 66 six-rowed accessions were used in this study. All accessions were planted in Changxing (30°55′ N, 119°47′ E) and Cixi (30°10′ N, 121°14′ E) of Zhejiang Province, China in early Nov. 2018. The soil in Changxing contained 1.22 g/kg total nitrogen, 8.9 mg/kg available phosphorus and 12.5 mg/kg available potassium, and pH was 6.0; while soil in Cixi contained 1.31 g/kg total nitrogen, 102 mg/kg available phosphorus and 163 mg/kg available potassium, and pH was 8.29. Each barley accession was planted in a plot consisting of five rows (2 m length and 0.25 m between rows). At maturity, grains in the plants of the middle three rows were harvested for further measurement. Other field managements, including fertilization, weed and disease control, were the same as used locally.

### 4.2. Measurement of Total Starch, Amylose and Amylopectin 

The harvested grains were dried, ground and passed through 30-mesh sieves for various measurements. The content of total starch, amylose/amylopectin were quantified using TOTAL STARCH kit (K-TSTA) and AMYLOSE/AMYLOPECTIN (K-AMYL) kits (Megazyme, Bray, Ireland) according to the manual instructions, respectively. Three biological replicates were performed for each measurement. 

### 4.3. SNP Markers, Population Structure and Kinship Analysis

Total RNA was isolated with RNAprep Pure Plant Kit (TIANGEN, Beijing, China) according to the manufacturer’s instructions. The cDNA library were constructed using reversed cDNA of every samples. The transcriptome librarys were sequenced using IIIumina HiSeq NOVA seq sequencing technology platform (Biomarker Technologies, Beijing, China). After qualitied control and the adapters, rRNAs and low quality reads removed, a total of 2.8Tb clean data were obtained for 100 BCS accessions. The Q30 values were all higher than 92%, with average value being 94.49%. The clean reads of transcriptome data were aligned with the Barley reference genome (http://plants.ensemble.org/ Hordeum_vulgare/info/Annotation/assembly) using TopHat 2.0.13 software [39]. The mapping rate was 73.47~80.93%. Then, the aligned results were sorted and filtered by SAMTOOLS 0.1.18 [40] and PICARD 1.94 software (http://broadinstitute.github.io/picard). The SNP mutations were identified with GATK 3.2-2 [41]. The filtering paraments were set as: -window 35 -cluster 3 -filterName FS-filter “FS > 30.0” -filterName QD -filter “QD < 2.0”. A total of 1,084,274 valid SNP data were obtained. Using a filter criteria of calling rate <0.85 and minor allele frequency <0.05, a total of 191,147 high-quality SNPs were obtained and used for population structure and kinship analysis as well as GWAS analysis. The population structure was investigated using ADMIXTURE (http://www.genetics.ucla.edu/software/admixture/) software. Cross-validation errors (CV errors) were calculated with K value from 1 to 7. The 100 BCS accessions were divided into optimal six subpopulations according to the lowest “CV error”. Tassel 5 (http://www.maizegenetica.net/tassel) was used to evaluate kinship based on Centered_IBS method. The LD parameter (r^2^) was calculated using plink software (http://www.cog-genomics.org/plink2). The LD decay plot was draw by R studio 1.2.1335. It was found the LD decay of 100 BCS population was approximately 100 kb. (Figure S1).

### 4.4. Genome-Wide Association Studies

In contrast with the general linear model (GLM) and mixed linear model (MLM) analysis (Figure S2), the QQ plots of efficient mixed-model association eXpedited (EMMAX) showed reliable results where the straight line is closed to the diagonal line but with a sharp upward deviated tail [23]. The improved model EMMAX was used to perform genome-wide association analysis [42], with the kinship (K) matrix as a random effect and population structure (Q) matrix as a fixed effect, respectively. Manhattan and quantile-quantile (QQ) plots for total starch, amylose and amylopectin were drawn by R studio 1.2.1335. The significance of SNP markers was determined by a threshold *p*-value of 10^−4^. To verify the accuracy of genotype data, the phenotype of naked caryopsis was analyzed firstly. The GWAS result showed that naked caryopsis of barley grain was significantly associated with *Nud*, the reported important gene in chromosome 7H (Figure S3) [43], proving the reliability of the analysis.

### 4.5. Statistical Analysis

The distribution frequency, correlation and haplotype analysis, and variance analysis were conducted using IBM SPSS Statistics 25 software (IBM, Armonk, NY, USA). The expression data were obtained from the BARLEX publicly available database and heatmap of candidate genes was drawn by TBtools [44] with the expression level (log_10_RPKM) of candidate genes. The genetic map was operated by using MapChart 2.2.0.0. (Plant Research International, Wageningen, Netherlands).

## Figures and Tables

**Table 1:** Phenotypic variation for TS, AC and AP in the association population.

Trait	Environment	Accessions	Mean	Max	Min	SD	CV(%)
TS	19CHX	97	64.31%	72.46%	51.76%	3.84%	5.97%
	19CX	97	59.55%	68.00%	50.36%	3.35%	5.63%
AC	19CHX	97	21.70%	30.73%	15.93%	3.31%	15.25%
	19CX	97	21.26%	25.24%	17.47%	1.63%	7.68%
AP	19CHX	97	42.61%	51.64%	30.47%	4.34%	10.18%
	19CX	97	38.29%	44.90%	32.16%	2.71%	7.09%

TS, total starch; AC, amylose; AP, amylopectin; SD, standard deviation; CV, coefficent of variation.

**Table 2:** The identified stable QTLs of TS, AC and AP in GWAS analysis.

Trait	QTL ID	SNP ID	Definition	Chr.	Position	Environments						Alleles	MAF
						2019 CHX		2019 CX		AVERAGE			
						(−logP)	R^2^ (%)	(−logP)	R^2^ (%)	(−logP)	R^2^ (%)		
TS	qTS-1.1	S1H_48897678		1	48897678			4.82	18.66%	5.20	19.96%	C/T	0.05
	qTS-1.2	S1H_534442471		1	534442471			5.13	20.52%	4.57	20.79%	G/A	0.185
	qTS-2.1	S2H_484808981	common QTL	2	484808981	5.17	21.87%	5.44	21.14%	6.68	26.01%	T/C	0.09
	qTS-3.1	S3H_176458677	common QTL/major QTL	3	176458677	5.42	24.00%	5.30	20.92%	6.69	27.15%	G/A	0.185
	qTS-3.2	S3H_541044868		3	541044868	5.40	22.45%	4.41	17.37%	6.18	24.62%	C/G	0.08
	qTS-4.1	S4H_506669263		4	506669263	5.84	25.80%	4.51	17.56%	6.52	26.65%	C/T	0.075
	qTS-5.1	S5H_536435763		5	536435763	5.80	25.48%			6.12	25.26%	G/A	0.075
	qTS-6.1	S6H_22809571		6	22809571	5.84	23.49%			5.04	20.44%	C/A	0.057
	qTS-6.2	S6H_349173230	common QTL	6	349173230	5.29	21.18%			4.71	18.60%	C/A	0.065
	qTS-6.3	S6H_469691793	common QTL	6	469691793	5.79	25.03%	4.47	17.52%	6.50	25.98%	C/A	0.14
	qTS-7.1	S7H_26188860	common QTL	7	26188860			4.16	16.68%	4.24	19.98%	C/T	0.055
	qTS-7.2	S7H_310869861		7	310869861	5.41	20.73%			4.93	18.90%	C/T	0.075
	qTS-7.3	S7H_637563550		7	637563550	4.12	21.01%			5.04	22.72%	G/A	0.155
AC	qAC-6.1	S6H_70242665		6	70242665			5.06	19.83%	4.08	15.10%	A/G	0.3
	qAC-6.2	S6H_565362485	major QTL	6	565362485	4.84	19.40%			5.00	20.65%	G/A	0.107
AP	qAP-2.1	S2H_484808981	common QTL	2	484808981	5.26	21.46%			5.62	22.52%	T/C	0.09
	qAP-3.1	S3H_176458677	common QTL	3	176458677	5.25	21.16%			5.70	23.00%	G/A	0.185
	qAP-3.2	S3H_667803604		3	667803604	5.39	22.26%			4.77	19.61%	C/A	0.071
	qAP-4.1	S4H_29429921		4	29429921	4.38	19.73%			4.10	20.79%	A/G	0.49
	qAP-5.1	S5H_29297679	major QTL	5	29297679	5.95	23.52%	4.00	15.81%	6.50	25.70%	G/A	0.075
	qAP-5.2	S5H_551372936		5	551372936			4.79	16.19%	5.31	17.03%	G/T	0.082
	qAP-6.1	S6H_4816646		6	4816646	4.15	18.35%	4.42	17.23%	5.32	21.76%	G/T	0.055
	qAP-6.2	S6H_349173230	common QTL	6	349173230	4.24	16.34%			4.08	15.57%	C/A	0.065
	qAP-6.3	S6H_469691793	common QTL	6	469691793	5.18	21.92%			4.82	20.23%	C/A	0.14
	qAP-7.1	S7H_26188860	common QTL	7	26188860			5.08	20.20%	4.97	21.25%	C/T	0.055

## Data Availability

Data is contained within the article or supplementary material.

## Supplementary Materials

The following are available online at https://www.mdpi.com/1422-0067/22/2/553/s1, Figure S1: Analysis of the population structure and kinship of 100 barley accessions, Figure S2: The comparison of QQ plots of general linear model (GLM), mixed linear model (MLM) analysis and efficient mixed-model association eXpedited (EMMAX) model among three environments of different traits, Figure S3: GWAS results and analysis of the significant peaks associated with naked caryopsis traits. Manhattan plot (b) and QQ plot (a) for naked caryopsis trait, Figure S4: GWAS results for total starch content and amylose trait. Manhattan plot (b) and QQ plot (a) in 2019, changxing. Manhattan plot (d) and QQ plot (c) in 2019, cixi. Manhattan plot (f) and QQ plot (e) of the mean phenotypic data between two environments, Figure S5: Haplotypes and Phenotypic differences of TS and AP content based on five common QTLs, Figure S6: The physical location (Mb) of stable QTLs and the known barley homologous genes with identified rice starch-associated genes on the barley genetic map, Table S1: Morphology, origin and population structure information for 100 barley core collection accessions, the total starch, amylose and amylopectin content (%) in different environments were measured, Table S2: Analysis of variance of total starch, amylose and amylopectin content in 100 barley accessions in three environments, Table S3: Single-nucleotide polymorphism marker information across all seven barley chromosomes and genetic diversity among the 100 barley accessions, Table S4: The significant QTLs detected in three environments of each traits, Table S5: Expression patterns of 15 candidate genes at different developmental stages and tissues, Table S6: Haplotypes of HORVU5Hr1G011270 observed in 100 BCS accessions with 11 SNPs, Table S7: Haplotypes of HORVU5Hr1G011280 observed in 100 BCS accessions with 5 SNPs, Table S8: The favorable alleles of identified QTLs for total starch, amylose, and amylopectin content of barley grains.
