# Supplementary material for: Genome-Wide Association Study on Total Starch, Amylose and Amylopectin in Barley Grain Reveals Novel Putative Alleles
Source: Int J Mol Sci. 2021 Jan 7;22(2):553. doi: 10.3390/ijms22020553 (PMC7828029; doi:10.3390/ijms22020553)
Supplement: Supplementary file 1 [file ijms-22-00553-s001.zip › suppl + table suppl revised/Supporting figures.docx]

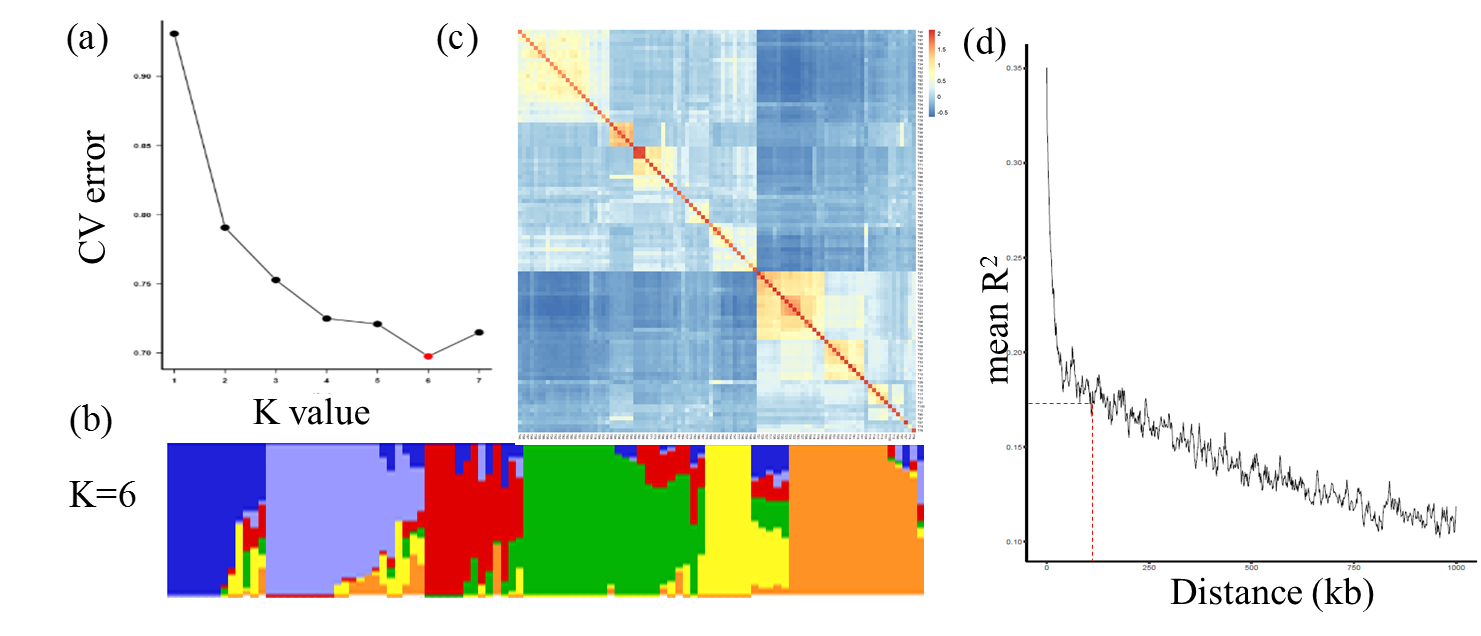


Figure S1 Analysis of the population structure and kinship of 100 barley accessions. (a) Cross-validation (CV) error change with the successive K. The red solid dot indicated the optimal subpopulations. (b) Population structure based on ADMIXTURE when K=6, the optimal subpopulation. (c) Kinship between the 100 accessions. (d) Genome-wide average LD decay estimated in the 100 BCS population.


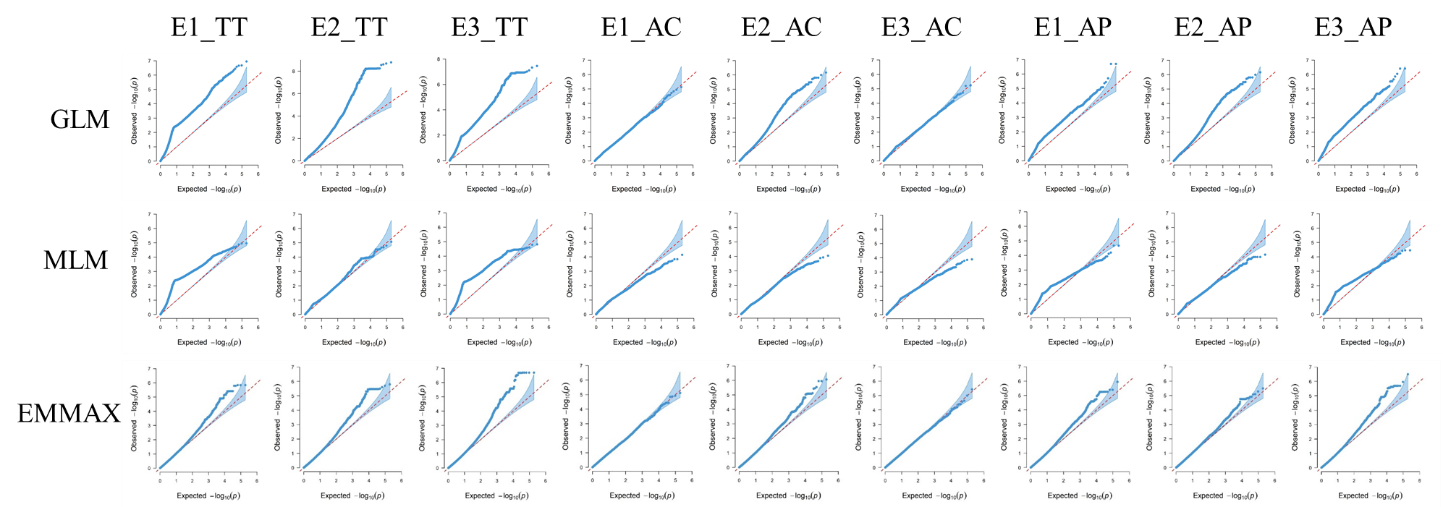


Figure S2 The comparison of QQ plots of general linear model (GLM), mixed linear model (MLM) analysis and efficient mixed-model association eXpedited (EMMAX) model among three environments of different traits.


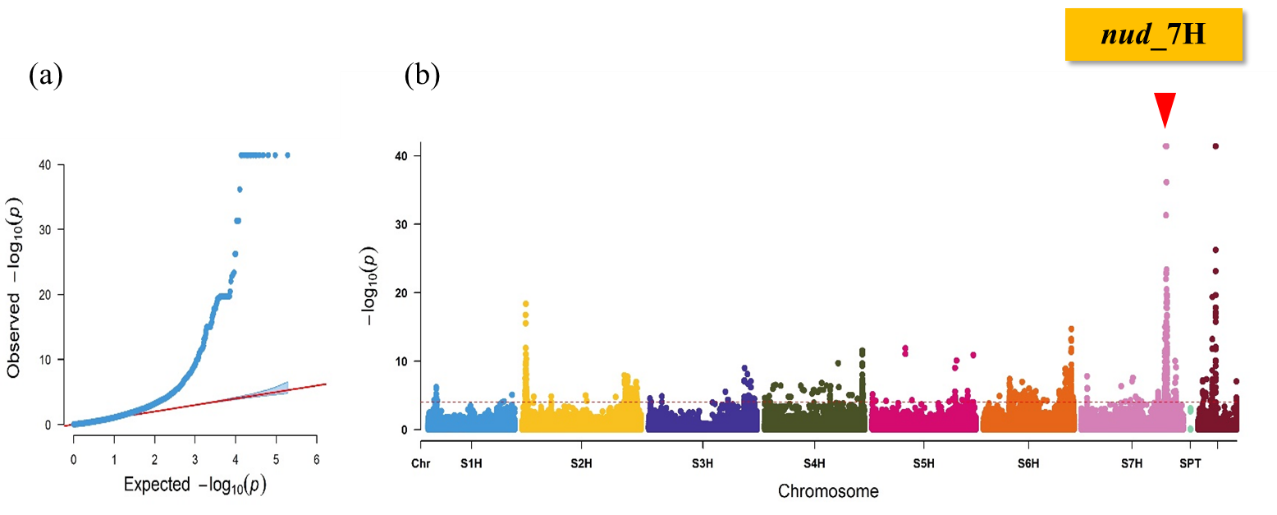


Figure S3 GWAS results and analysis of the significant peaks associated with naked caryopsis traits. Manhattan plot (b) and QQ plot (a) for naked caryopsis trait. The dashed line represents the significance threshold (P < 10^-4^). The red arrowhead indicates the position of the strong peak on chromosome 7H investigated in this study.


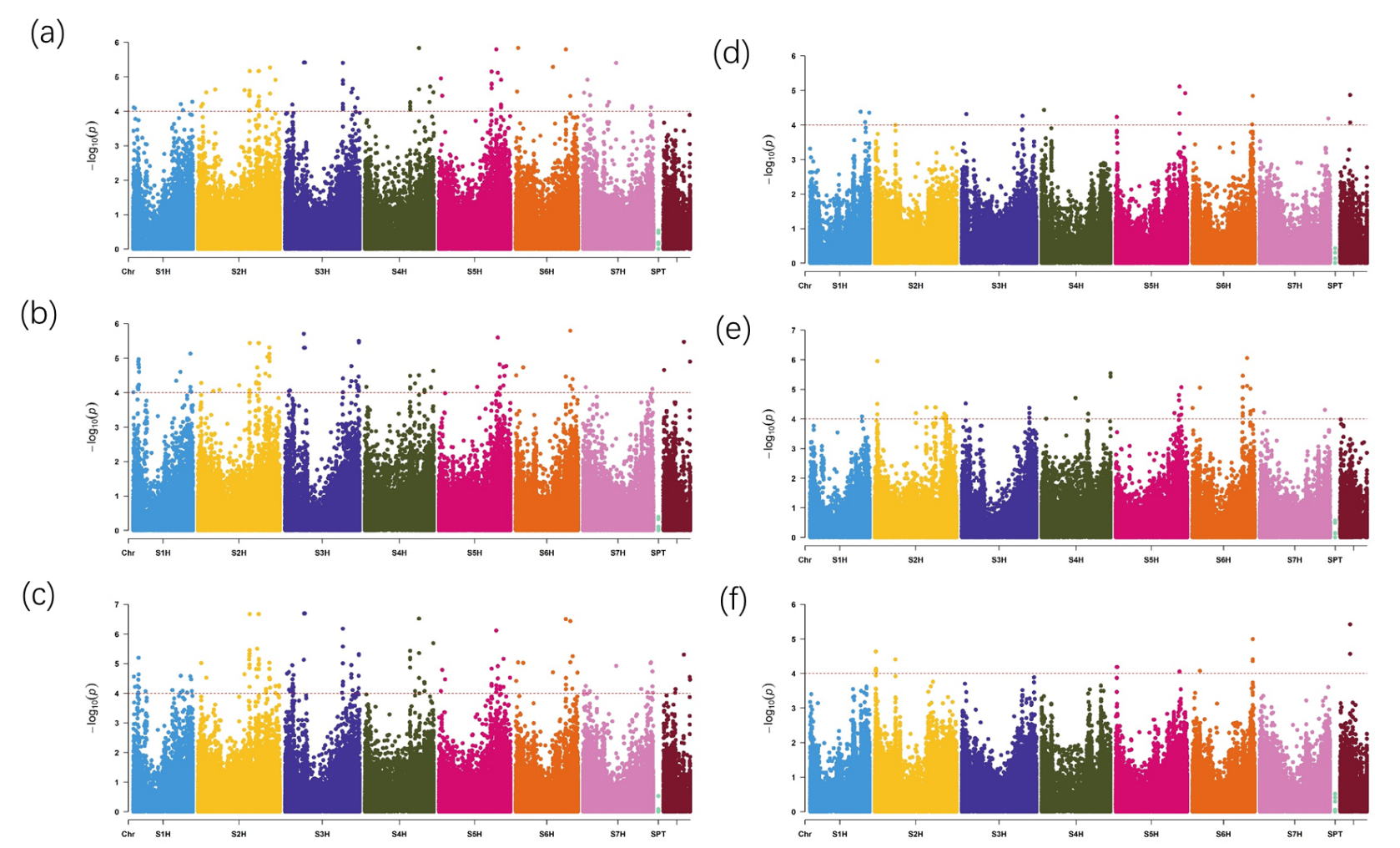


Figure S4 GWAS results for total starch content and amylose trait. Manhattan plot (b) and QQ plot (a) in 2019, changxing. Manhattan plot (d) and QQ plot (c) in 2019, cixi. Manhattan plot (f) and QQ plot (e) of the mean phenotypic data between two environments. Dashed line represents the significance threshold, respectively (P < 10-4).


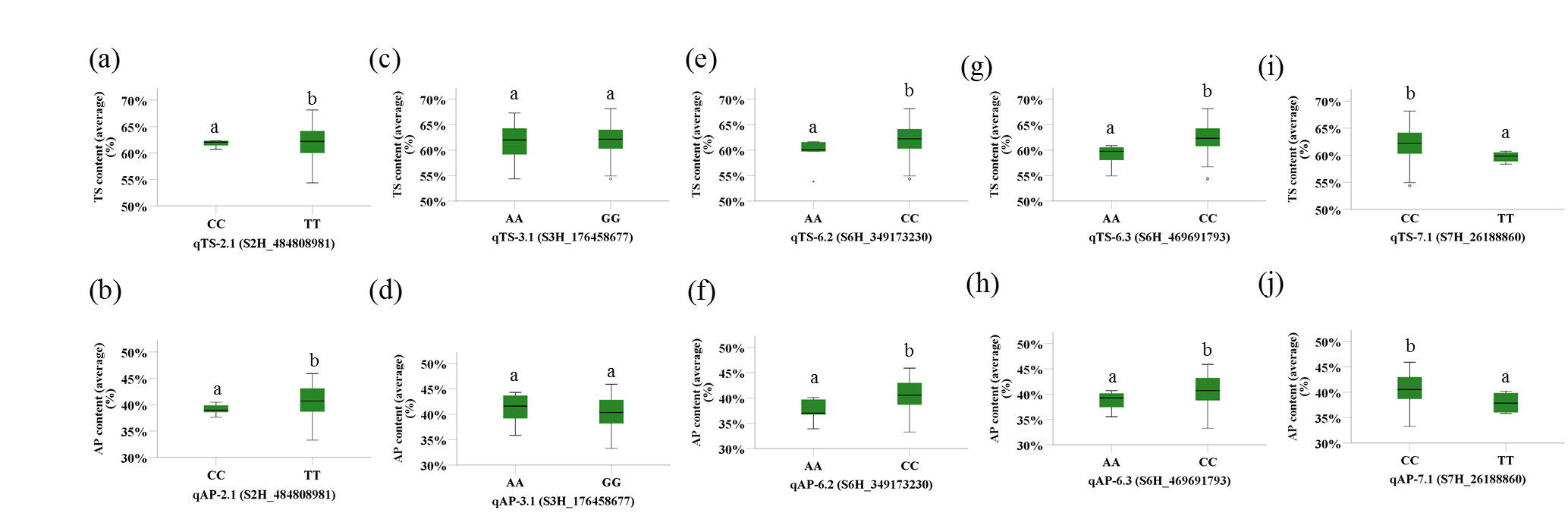


Figure S5 Haplotypes and Phenotypic differences of TS and AP content based on five common QTL. (a, b) qTS-2.1 (qAP-2.1, S2H_484808981), (c, d) qTS-3.1 (qAP-3.1, S3H_176458677), (e, f) qTS-6.2 (qAP-6.2, S6H_349173230), (g, h) qTS-6.3 (qAP-6.3, S6H_469691793) and (i, j) qTS-7.1 (qAP-7.1, S7H_26188860). Different letters represent the significant differences (p<0.05).


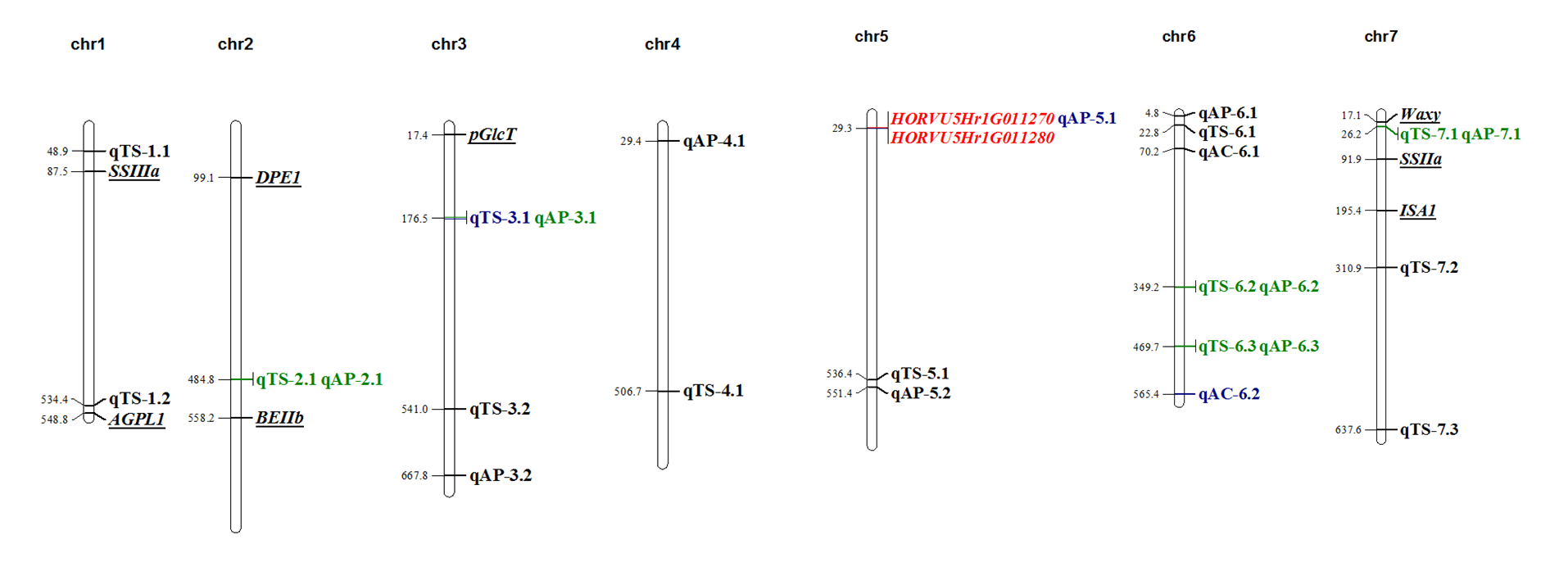


Figure S6 The physical location (Mb) of stable QTL and the known barley homologous genes with identified rice starch-associated genes on the barley genetic map. The location of *SSIIIa*, *AGPL1*, *DPE1*, *pGlcT*, *ISA1*, *Waxy, SSIIa* and *BEIIb* was identified based on the homology with rice. The common QTL and major QTL were highlight with green and blue color, respectively. The candidate genes were highlight with red color.
